# Exact conditions for preservation of the partial indices of a perturbed triangular 2 × 2 matrix function

**DOI:** 10.1098/rspa.2020.0099

**Published:** 2020-05-27

**Authors:** Victor M. Adukov, Gennady Mishuris, Sergei V. Rogosin

**Affiliations:** 1 Institute of Natural Sciences and Mathematics, South Ural State University, 454080 Chelyabinsk, Russia; 2 Institute of Mathematics, Physics and Computer Science, Aberystwyth University, Aberystwyth SY23 3BZ, UK; 3 Department of Economics, Belarusian State University, 220030 Minsk, Belarus

**Keywords:** factorization of matrix functions, triangular matrices, Wiener algebra, Toeplitz matrix, essential polynomials

## Abstract

The possible instability of partial indices is one of the important constraints in the creation of approximate methods for the factorization of matrix functions. This paper is devoted to a study of a specific class of triangular matrix functions given on the unit circle with a stable and unstable set of partial indices. Exact conditions are derived that guarantee a preservation of the unstable set of partial indices during a perturbation of a matrix within the class. Thus, even in this probably simplest of cases, when the factorization technique is well developed, the structure of the parametric space (guiding the types of matrix perturbations) is non-trivial.

## 1. Introduction

In the classical framework, the (right) factorization problem involves the representation of a square non-singular matrix function $G\in G{\left(M(\Gamma )\right)}^{n\times n}$, defined on a simple closed smooth curve *Γ* in the complex plane C, in the following form:

1.1 $$G(t)=G^{-}(t)\Lambda (t)G^{+}(t).$$

Here, non-singular matrices *G*^−^(*t*), *G*^+^(*t*) possess, together with their inverses, analytic continuations into *D*^−^ and *D*^+^, respectively, where *D*^−^, *D*^+^ are the domains on the Riemann sphere lying, respectively, to the right and to the left of the curve *Γ*, with reference to the orientation chosen for *Γ*. Finally, *Λ*(*t*) is the *n* × *n* diagonal matrix,

1.2 $$\Lambda (t)=\text{diag}\left\{{\left(\frac{t-t^{+}}{t-t^{-}}\right)}^{\rho_{1}},\ldots ,{\left(\frac{t-t^{+}}{t-t^{-}}\right)}^{\rho_{n}}\right\},$$

where $\rho_{j}\in Z$ are the so-called partial indices and *t*^+^ ∈ *D*^+^, *t*^−^ ∈ *D*^−^ are certain (fixed) points.

In particular, when Γ=T (that is, the unit circle on the complex plane), the diagonal matrix *Λ*(*t*) takes the form

1.3 $$\Lambda (t)=\text{diag}\{t^{\rho_{1}},\ldots ,t^{\rho_{n}}\}.$$

Factorization plays an important role in the study of many applied problems (see [1,2]). In the one-dimensional case, the factorization problem possesses a complete and explicit solution [3]. In the matrix case (*n* > 1), some explicit methods of factorization have been found only for a few classes of matrices (among these, we point out functionally commutative matrices [4], triangular matrices with factorizable diagonal elements [5,6], rational matrices [7] and a few more special classes of matrix functions (e.g. [8])). For extended information on the available explicit factorizations, see [2,9] and references therein. Therefore, several attempts have been made to find approximating procedures. An essential constraint in this respect was found independently by Gohberg & Krein [10] and by Bojarski [11]. They introduced the notion of a *stable set of partial indices* for a non-singular matrix function (those that preserve their values with a small perturbation of the matrix). A criterion for the stability was evaluated, specifically max*ρ*_*j*_ − min*ρ*_*j*_ ≤ 1, and recipes were proposed on how, under the condition max*ρ*_*j*_ − min*ρ*_*j*_ > 1, one might construct another matrix with a different set of partial indices that lies in an arbitrarily small neighbourhood of a given matrix (see also [11,12]). The above stability criterion is usually called the Gohberg–Krein–Bojarski (GKB) criterion.

The main differences between the scalar and matrix Wiener–Hopf factorization problems are the following: an explicit factorization is not always possible even for fairly simple matrix functions; the total index ϰ=inddetG(t) remains the same under a small perturbation with respect to a certain matrix norm, but this is not the case for partial indices.

In general, partial indices are not preserved under even a small perturbation of a matrix *A*(*t*) if the GKB condition is not satisfied. However, if *A*(*t*) belongs to a certain subclass of the matrix functions and we consider perturbations only within that subclass, then the partial indices can be common for all matrices sufficiently close to the original matrix function *A*(*t*).

We note that the partial indices can always be ordered such that either *ρ*_1_ ≤ *ρ*_2_ ≤ · · · ≤ *ρ*_*n*−1_ ≤ *ρ*_*n*_ or *ρ*_*n*_ ≤ *ρ*_*n*−1_ ≤ · · · ≤ *ρ*_2_ ≤ *ρ*_1_ [13]. The factors *G*^−^, *G*^+^ in (1.1) are determined up to a certain rational block triangular factorization (e.g. [14, theorem 1.2, ch. I], cf. [15, theorem 1.2]). From the above-mentioned theorem it follows, in particular, that the factorization can be made unique by applying a number of linear conditions; for details, see [9, theorem 1.4].^1^ One possible normalization has already been proposed by Riemann in his work on the construction of the differential equation with a prescribed group of monodromy (the so-called Riemann–Hilbert problem). Specifically, he required that *G*_−_(*z*) → *I* as *z* → ∞ inside *D*^−^ (cf. [15,16]), which guarantees the uniqueness of the factors in some cases.

A challenging problem in factorization theory is that the description of a path (surface) in the parametric space preserving an unstable set of partial indices remains unsolved. The study of this problem was initiated by Bojarski [11]. He demonstrated that the set of matrices with the same partial indices is in general not an open set in the class of invertible matrix functions with a given order of smoothness, but is still a connected set. It was also shown that the only open set constitutes the matrices with fixed stable collections of partial indices. Bojarski [17] also introduced the notion of the homotopic equivalence of matrices, i.e. those connected by a continuous collection of invertible matrix functions, and formulated the above results in terms of such a notion. In [18], a class of perturbations of a matrix with unstable partial indices, which are preserved under the specific perturbation, was studied and constructive, although rather cumbersome, conditions were established (see also [19]). For an arbitrary matrix function, the description of possible perturbations that preserve their partial indices remains uncovered.

The aim of the present paper is to analyse the perturbations in a class of 2 × 2 triangular matrix functions. On one hand, such matrices are among the simpler examples, which also include the diagonal matrices, and their factorizations can be obtained explicitly (see [13,20–23]), while, as can be seen from the following, the results are not at all trivial. On the other hand, triangular matrices play a crucial role in the factorization of a matrix function of general form [24].

In our paper for a given triangular 2 × 2 matrix function *A*(*t*), we consider only a set of *triangular* matrix functions, $\tilde{A}(t)$, close to the original one, $||A(t)-\tilde{A}(t)||<\varepsilon$. In the GKB criterion, arbitrary (not only triangular) perturbed matrix functions are analysed. In this way, we have established new stability cases that do not obey the GKB criterion. Moreover, our criterion is effective as it simultaneously determines the partial indices. However, unsurprisingly, in most cases, our criterion coincides with the classic GKB criterion.

The paper is organized as follows. Section 2 introduces the main notations and presents important preliminary results. Section 3 is devoted to the construction of the solution of the factorization problem for a different relationship between the indices *ν*_1_, *ν*_2_ of the diagonal elements of the given matrix of the above class. In §4, we study the stability of the set of partial indices in their dependence on *ν*_1_, *ν*_2_ and of the perturbation of the given matrix. Rigorous proofs for some of the statements are given in appendix A.

## 2. Notations and preliminary results

Let us denote the unit circle by T={z∈C: |z|=1}. The *Wiener algebra* W(T) is the set of all functions represented by the absolutely converging Fourier series,

$$f(t)=\sum_{j=-\infty }^{\infty }f_{j}t^{j},\;t\in T,$$

which is equipped with the standard norm $||f||:=\sum_{j=-\infty }^{\infty }|f_{j}|<\infty$. Thus W(T) is the Banach algebra [9, sec. 2]. The sets

$$W_{+}(T):=\{f\in W:f_{j}=0,j<0\},\;\;W_{-}(T):=\{f\in W:f_{j}=0,j\geq 0\}$$

are closed subalgebras of W(T) , such that W(T) may be decomposed: $W(T)=W_{-}(T)⊕W_{+}(T)$. Thus, any invertible elements of W(T) admit the Wiener–Hopf factorization [9, sec. 2, cor. 2.13].

The algebra of 2 × 2 matrix functions with entries *a*_*ij*_(*t*) in W(T) will be denoted by $W^{2\times 2}(T)$. This is a Banach algebra with respect to one of the usual matrix norms, e.g.

$$||A(t)||=\max\{||a_{11}(t)||+||a_{21}(t)||,||a_{21}(t)||+||a_{22}(t)||\}$$

and $W_{-}^{2\times 2}(T)$, $W_{+}^{2\times 2}(T)$ are its closed subalgebras. $GW^{2\times 2}(T)$ is the group of all invertible elements from $W^{2\times 2}(T)$. Any $A(t)\in GW^{2\times 2}(T)$ admits the Wiener–Hopf factorization in this algebra.

Let us denote by $TW^{2\times 2}(T)$ a class of triangular 2 × 2 matrix functions

$$A(t)=\left(\begin{array}{cc} a_{11}(t) & 0\\ a_{21}(t) & a_{22}(t) \end{array}\right)$$

with entries *a*_*ij*_(*t*) in W(T). This is a closed subalgebra of $W^{2\times 2}(T)$. We denote the group of all invertible elements from $TW^{2\times 2}(T)$ by $GTW^{2\times 2}(T)$. In general, $A(t)\in GTW^{2\times 2}(T)$ admits the Wiener–Hopf factorization in $GW^{2\times 2}(T)$, but the factors are not necessarily triangular matrices; see [5].

For any $A(t)\in GTW^{2\times 2}(T)$, the factorizations of the diagonal elements are known, $a_{jj}(t)=a_{jj}^{-}(t)t^{\nu_{j}}a_{jj}^{+}(t)$, *ν*_*j*_ = ind *a*_*jj*_(*t*), and such factorizations are unique provided that $a_{jj}^{+}(0)=1$.

Let

2.1 $$a_{j}=\frac{1}{2\pi i}\int_{|t|=1}t^{-j-1}\frac{a_{21}(t)}{a_{11}^{+}(t)a_{22}^{-}(t)}\;\text{d}t$$

be the Fourier coefficients of the function

2.2 $$a(t)=\frac{a_{21}(t)}{a_{11}^{+}(t)a_{22}^{-}(t)}.$$

Since we deal only with matrix functions defined on the unit circle T, we will use the shorter notations $GW^{2\times 2}$, $TW^{2\times 2}$, $GTW^{2\times 2}$ etc. for the corresponding classes of matrix functions.

## 3. Relationship between the partial indices of a matrix function $GTW^{2\times 2}$ and the indices of its diagonal elements

Here, we explicitly solve the Wiener–Hopf factorization problem for the class $GTW^{2\times 2}$. A relationship is established between the partial indices *ρ*_1_, *ρ*_2_ of the matrix function *A*(*t*) and the indices *ν*_1_, *ν*_2_ of its diagonal elements.

**Remark 3.1.**

As mentioned in the Introduction, it is always possible to rearrange the order of partial indices in a delivered factorization to guarantee the condition *ρ*_1_ ≥ *ρ*_2_. We can directly check that the transformation $A(s)=A_{-}(s)D_{\rho_{1},\rho_{2}}(s)A_{+}(s)$ = $A_{-}(s)J\cdot JD_{\rho_{1},\rho_{2}}(s)J\cdot JA_{+}(s)$ = $A_{-}(s)J\cdot D_{\rho_{2},\rho_{1}}(s)\cdot JA_{+}(s)$ rearranges the initial factorization to that with the opposing order of partial indices. Here

$$J=\left(\begin{array}{cc} 0 & 1\\ 1 & 0 \end{array}\right),\;\text{and}\;J^{2}=I.$$

Taking this into account and noting that the factors in a factorization are not defined uniquely, below we will not pay any attention to the particular order of the partial indices in a delivered factorization.

To construct a factorization of *A*(*t*), we distinguish two cases (see §3a,b), since they require different techniques in analysis.

### (a) The case of *ν*_2_ ≤ *ν*_1_ + 1

The factorization in this case can be constructed following the method reported, for example, in [5]. Indeed, let us represent *A*(*t*) in the form

3.1 $$A(t)=\left(\begin{array}{cc} a_{11}^{-}(t) & 0\\ 0 & a_{22}^{-}(t) \end{array}\right)A_{0}(t)\left(\begin{array}{cc} a_{11}^{+}(t) & 0\\ 0 & a_{22}^{+}(t) \end{array}\right),$$

where

3.2 $$A_{0}(t)=\left(\begin{array}{cc} t^{\nu_{1}} & 0\\ a(t) & t^{\nu_{2}} \end{array}\right)$$

and *a*(*t*) is defined by formula (2.2). Now let us split the function *a*(*t*) into

$$a(t)=\sum_{j=-\infty }^{\zeta }a_{j}t^{j}+\sum_{j=\zeta +1}^{\infty }a_{j}t^{j},$$

where *ν*_2_ − 1 ≤ *ζ* ≤ *ν*_1_. Then

3.3 $$A_{0}(t)=\left(\begin{array}{cc} 1 & 0\\ t^{-\nu_{1}}\sum_{j=-\infty }^{\;\zeta }a_{j}t^{j} & 1 \end{array}\right)\left(\begin{array}{cc} t^{\nu_{1}} & 0\\ 0 & t^{\nu_{2}} \end{array}\right)\left(\begin{array}{cc} 1 & 0\\ t^{-\nu_{2}}\sum_{j=\zeta +1}^{\infty }a_{j}t^{j} & 1 \end{array}\right)$$

is the Wiener–Hopf factorization of *A*_0_(*t*), since the skew-diagonal elements of the matrices are

$$t^{-\nu_{1}}\sum_{j=-\infty }^{\zeta }a_{j}t^{j}\in W_{-}(T),\;\;t^{-\nu_{2}}\sum_{j=\zeta +1}^{\infty }a_{j}t^{j}\in W_{+}(T).$$

By (3.1)–(3.3) we obtain a factorization of *A*(*t*) with partial indices *ρ*_1_ = *ν*_1_, *ρ*_2_ = *ν*_2_. Let us consider three subcases that differ in their choice of *ζ*.

*First special case:* *ν*_1_ = *ν*_2_. Using the technique discussed above, we can construct two triangular factorizations, taking *ζ*_1_ = *ν*_1_ or *ζ*_2_ = *ν*_1_ − 1. We then have

3.4 $$A_{0}^{\left(k\right)}(t)=\left(\begin{array}{cc} 1 & 0\\ a_{-}^{\left(k\right)}(t) & 1 \end{array}\right)\left(\begin{array}{cc} t^{\nu_{1}} & 0\\ 0 & t^{\nu_{1}} \end{array}\right)\left(\begin{array}{cc} 1 & 0\\ a_{+}^{\left(k\right)}(t) & 1 \end{array}\right),\;k=1,2,$$

where

3.5 $$a_{-}^{\left(1\right)}(t)=t^{-\nu_{1}}\sum_{j=-\infty }^{\;\nu_{1}}a_{j}t^{j},\;a_{-}^{\left(2\right)}(t)=t^{-\nu_{1}}\sum_{j=-\infty }^{\;\nu_{1}-1}a_{j}t^{j},$$

and

3.6 $$a_{+}^{\left(1\right)}(t)=t^{-\nu_{1}}\sum_{j=\nu_{1}+1}^{\infty }a_{j}t^{j},\;a_{+}^{\left(2\right)}(t)=t^{-\nu_{1}}\sum_{j=\nu_{1}}^{\infty }a_{j}t^{j}.$$

*Second special case: *ν*_1_ = *ν*_2_ − 1.* In this case the only possible option for *ζ* is to choose *ζ* = *ν*_1_. We note that this case corresponds to the stable configuration of the partial indices (as well as to the previous special case). Now the matrix function *A*_0_(*t*) is factorized as follows:

3.7 $$A_{0}(t)=\left(\begin{array}{cc} 1 & 0\\ a_{-}(t) & 1 \end{array}\right)\left(\begin{array}{cc} t^{\nu_{1}} & 0\\ 0 & t^{\nu_{1}+1} \end{array}\right)\left(\begin{array}{cc} 1 & 0\\ a_{+}(t) & 1 \end{array}\right),$$

where

$$a_{-}(t)=t^{-\nu_{1}}\sum_{j=-\infty }^{\nu_{1}}a_{j}t^{j},\;a_{+}(t)=t^{-\nu_{1}-1}\sum_{j=\nu_{1}+1}^{+\infty }a_{j}t^{j}.$$

*Third special case: *ν*_1_ − *ν*_2_ + 1 = *s* > 0.* In this case, there are exactly *s* + 1 choices for *ζ*. Thus we have *s* + 1 formulae for factorization, specifically

3.8 $$A_{0}^{\left(k\right)}(t)=\left(\begin{array}{cc} 1 & 0\\ a_{-}^{\left(k\right)}(t) & 1 \end{array}\right)\left(\begin{array}{cc} t^{\nu_{1}} & 0\\ 0 & t^{\nu_{2}} \end{array}\right)\left(\begin{array}{cc} 1 & 0\\ a_{+}^{\left(k\right)}(t) & 1 \end{array}\right),\;k=0,1,\ldots ,s,$$

where

3.9 $$a_{-}^{\left(k\right)}(t)=t^{-\nu_{1}}\sum_{j=-\infty }^{\;\nu_{2}-1+k}a_{j}t^{j},\;a_{+}^{\left(k\right)}(t)=t^{-\nu_{2}}\sum_{j=\nu_{2}+k}^{+\infty }a_{j}t^{j}.$$

The partial indices in this case coincide with the orders of the diagonal elements, *ρ*_1_ = *ν*_1_, *ρ*_2_ = *ν*_2_ = *ν*_1_ + 1 − *s*.

**Example 3.2.**

Let

$$A_{0}(t)=\left(\begin{array}{cc} t^{2} & 0\\ t^{-3}-2t^{-1}+3-4t+t^{2}+t^{5} & t^{-2} \end{array}\right),\;\nu_{1}=2,\;\nu_{2}=-2.$$

Then one of the possible representations (3.8) for *k* = *s*,

$$A_{0}^{\left(5\right)}(t)=\left(\begin{array}{cc} 1 & 0\\ t^{-5}-2t^{-3}+3t^{-2}-4t^{-1}+1 & 1 \end{array}\right)\left(\begin{array}{cc} t^{2} & 0\\ 0 & t^{-2} \end{array}\right)\left(\begin{array}{cc} 1 & 0\\ t^{7} & 1 \end{array}\right),$$

is the Wiener–Hopf factorization of *A*_0_(*t*).

### (b) The case of *ν*_2_ ≥ *ν*_1_ + 2

We split this case into two subcases, as follows.

(i) $a_{\nu_{1}+1}=\cdots =a_{\nu_{2}-1}=0.$ Then in formula (3.3) we obtain

$$t^{-\nu_{2}}\sum_{j=\nu_{1}+1}^{\infty }a_{j}t^{j}=t^{-\nu_{2}}\sum_{j=\nu_{2}}^{\infty }a_{j}t^{j}\in W_{+}$$

and (3.3) gives the factorization of *A*_0_(*t*). The factorization of *A*(*t*) is constructed by formulae (3.1)–(3.3) and the partial indices are the same as those previously determined, *ρ*_1_ = *ν*_1_, *ρ*_2_ = *ν*_2_.

**Example 3.3.**

Let

$$A_{0}(t)=\left(\begin{array}{cc} t^{-2} & 0\\ t^{-3}+t^{2}+t^{5} & t^{2} \end{array}\right),\;\nu_{1}=-2,\;\nu_{2}=2,\;a_{-1}=a_{0}=a_{1}=0.$$

Then the representation

$$A_{0}(t)=\left(\begin{array}{cc} 1 & 0\\ t^{-1} & 1 \end{array}\right)\left(\begin{array}{cc} t^{-2} & 0\\ 0 & t^{2} \end{array}\right)\left(\begin{array}{cc} 1 & 0\\ 1+t^{3} & 1 \end{array}\right)$$

is the Wiener–Hopf factorization of *A*_0_(*t*).

(ii) The sequence $a_{\nu_{1}+1}^{\nu_{2}-1}:=\{a_{\nu_{1}+1},\ldots ,a_{\nu_{2}-1}\}$ is non-zero. Let us represent *A*_0_(*t*) by the following:

3.10 $$A_{0}(t)=\left(\begin{array}{cc} t^{\nu_{1}} & 0\\ a(t) & t^{\nu_{2}} \end{array}\right)=\left(\begin{array}{cc} 1 & 0\\ t^{-\nu_{1}}\sum_{j=-\infty }^{\nu_{1}}a_{j}t^{j} & 1 \end{array}\right)\left(\begin{array}{cc} t^{\nu_{1}} & 0\\ \sum_{j=\nu_{1}+1}^{\nu_{2}-1}a_{j}t^{j} & t^{\nu_{2}} \end{array}\right)\left(\begin{array}{cc} 1 & 0\\ t^{-\nu_{2}}\sum_{j=\nu_{2}}^{\infty }a_{j}t^{j} & 1 \end{array}\right).$$

Here the left factor belongs to $W_{-}^{2\times 2}$, and the right factor belongs to $W_{+}^{2\times 2}$. Hence the factorization is reduced to the factorization of the special rational triangular matrix function

3.11 $$P(t)=\left(\begin{array}{cc} t^{\nu_{1}} & 0\\ \sum_{j=\nu_{1}+1}^{\nu_{2}-1}a_{j}t^{j} & t^{\nu_{2}} \end{array}\right).$$

Let *ν*_1_ + 1 = *ν*_2_ − 1, that is, *ν*_2_ = *ν*_1_ + 2 and $a_{\nu_{1}+1}^{\nu_{2}-1}$ is a one-term sequence, i.e. $a_{\nu_{1}+1}\neq 0$. Then

$$P(t)=t^{\nu_{1}+1}\left(\begin{array}{cc} t^{-1} & 0\\ a_{\nu_{1}+1} & t \end{array}\right).$$

The factorization of P(t) can then be constructed directly

3.12 $$\begin{array}{rl} P(t) & =t^{\nu_{1}+1}\left(\begin{array}{cc} t^{-1} & -a_{\nu_{1}+1}^{-1}\\ a_{\nu_{1}+1} & 0 \end{array}\right)\left(\begin{array}{cc} 1 & a_{\nu_{1}+1}^{-1}t\\ 0 & 1 \end{array}\right)\\ & =\left(\begin{array}{cc} t^{-1} & -a_{\nu_{1}+1}^{-1}\\ a_{\nu_{1}+1} & 0 \end{array}\right)\left(\begin{array}{cc} t^{\nu_{1}+1} & 0\\ 0 & t^{\nu_{1}+1} \end{array}\right)\left(\begin{array}{cc} 1 & a_{\nu_{1}+1}^{-1}t\\ 0 & 1 \end{array}\right). \end{array}$$

Thus, in this case, the factorization of *A*(*t*) is constructed by formulae (3.1)–(3.12) and *ρ*_1_ = *ν*_1_ + 1, *ρ*_2_ = *ν*_1_ + 1.

**Example 3.4.**

Let

$$A_{0}(t)=\left(\begin{array}{cc} t^{} & 0\\ t^{-3}-2t^{-1}+3-4t+2t^{2}+t^{5}-t^{6} & t^{3} \end{array}\right),\;\;\nu_{1}=1,\;\nu_{2}=3,\;a_{2}=2\neq 0.$$

Then

$$A_{0}(t)=\left(\begin{array}{cc} 1 & 0\\ t^{-4}-2t^{-2}+3t^{-1}-4 & 1 \end{array}\right)\left(\begin{array}{cc} t & 0\\ 2t^{2} & t^{3} \end{array}\right)\left(\begin{array}{cc} 1 & 0\\ t^{2}-t^{3} & 1 \end{array}\right).$$

If we factor the middle multiplier by formula (3.12), we obtain the Wiener–Hopf factorization of *A*_0_(*t*),

$$\begin{array}{rl} A_{0}(t) & =\left(\begin{array}{cc} t^{-1} & -\frac{1}{2}\\ t^{-5}-2t^{-3}+3t^{-2}-4t^{-1}+2 & -\frac{1}{2}t^{-4}+t^{-2}-\frac{3}{2}t^{-1}+2 \end{array}\right)\left(\begin{array}{cc} t^{2} & 0\\ 0 & t^{2} \end{array}\right)\\ & \;\times \left(\begin{array}{cc} 1+\frac{1}{2}t^{2}-\frac{1}{2}t^{4} & \frac{1}{2}t\\ t^{2}-t^{3} & 1 \end{array}\right). \end{array}$$

A non-trivial case arises when the non-zero sequence $a_{\nu_{1}+1}^{\nu_{2}-1}:=\{a_{\nu_{1}+1},\ldots ,a_{\nu_{2}-1}\}$ consists of more than one term, that is, for *ν*_2_ ≥ *ν*_1_ + 3. The factorization of P(t) now requires a special technique, which is presented in appendix A.

Let *M* = *ν*_1_ + 1, *N* = *ν*_2_ − 1 and [(*M* + *N*)/2] = [(*ν*_1_ + *ν*_2_)/2] = *ν*, where

$$\nu :=\left\{ \begin{array}{l} \frac{\nu_{1}+\nu_{2}}{2},\;\text{if}\;\nu_{1}+\nu_{2}\;\text{is even},\\ \frac{\nu_{1}+\nu_{2}-1}{2},\;\text{if}\;\nu_{1}+\nu_{2}\;\text{is odd}. \end{array}\right.$$

We recall that $\nu_{1}+\nu_{2}=\varkappa =\text{ind}\det A(t)$.

Let *μ*_1_, *μ*_2_ be the indices and *R*_1_(*t*), *R*_2_(*t*) be essential polynomials of the sequence $a_{\nu_{1}+1}^{\nu_{2}-1}=a_{M}^{N}$ (see appendix A(a)).

By proposition .4, the indices of the sequence $a_{\nu_{1}+1}^{\nu_{2}-1}$ can be found by the formula

$$\mu_{1}=\nu_{1}+\rho ,\;\mu_{2}=\nu_{2}-\rho .$$

Here *ρ* is equal to the rank of the (*ν*_2_ − *ν*) × (*ν* − *ν*_1_) Toeplitz matrix $T_{\nu }=T_{\nu }(a_{M}^{N})$. For ϰ=2ν, the matrix *T*_*ν*_ is the square (*ν* − *ν*_1_) × (*ν* − *ν*_1_) matrix

3.13 $$T_{\nu }=\left(\begin{array}{ccc} a_{\nu } & \ldots & a_{\nu_{1}+1}\\ \vdots & \ddots & \vdots \\ a_{\nu_{2}-1} & \ldots & a_{\nu } \end{array}\right),$$

and for ϰ=2ν+1 it is the rectangular (*ν* − *ν*_1_ + 1) × (*ν* − *ν*_1_) matrix

3.14 $$T_{\nu }=\left(\begin{array}{ccc} a_{\nu } & \ldots & a_{\nu_{1}+1}\\ \vdots & \ddots & \vdots \\ a_{\nu_{2}-2} & \ldots & a_{\nu }\\ a_{\nu_{2}-1} & \ldots & a_{\nu +1} \end{array}\right).$$

We denote by $a_{M}^{N}(t)=\sum_{j=M}^{N}a_{j}t^{j}$ the generating function of the sequence $a_{M}^{N}$ and present the function $a_{\nu_{1}+1}^{\nu_{2}-1}(t)R_{j}(t)$ in the following way:

$$a_{\nu_{1}+1}^{\nu_{2}-1}(t)R_{j}(t)=t^{\mu_{j}}\alpha_{j}^{-}(t)-t^{\nu_{2}}\beta_{j}^{+}(t),\;j=1,2$$

(see appendix A(b), equation (A 10)).

Then the Wiener–Hopf factorization of P(t) can be found via the following formula (see appendix A(c), equation (A 12)):

3.15 $$P(t)=\sigma_{0}^{-1}\left(\begin{array}{cc} R_{1}^{-}(t) & R_{2}^{-}(t)\\ \alpha_{1}^{-}(t) & \alpha_{2}^{-}(t) \end{array}\right)\left(\begin{array}{cc} t^{\nu_{1}+\rho } & 0\\ 0 & t^{\nu_{2}-\rho } \end{array}\right)\left(\begin{array}{cc} \beta_{2}^{+}(t) & -R_{2}(t)\\ -\beta_{1}^{+}(t) & R_{1}(t) \end{array}\right),\;t\in T.$$

Now the Wiener–Hopf factorization of the original triangular matrix function *A*(*t*) is constructed via formulae (3.1), (3.10) and (3.15) with the right partial indices *ρ*_1_ = *ν*_1_ + *ρ*, *ρ*_2_ = *ν*_2_ − *ρ*.

A final result concerning the calculation of the right partial indices *ρ*_1_, *ρ*_2_ of a triangular 2 × 2 matrix function can be formulated into the following theorem.

**Theorem 3.5.**

*Let*

$$A(t)=\left(\begin{array}{cc} a_{11}(t) & 0\\ a_{21}(t) & a_{22}(t) \end{array}\right)\in TW^{2\times 2}$$

*be an invertible matrix function.*

*Suppose that*

$$a_{11}(t)=a_{11}^{-}(t)t^{\nu_{1}}a_{11}^{+}(t),\;a_{22}(t)=a_{22}^{-}(t)t^{\nu_{2}}a_{22}^{+}(t),\;a_{11}^{+}(0)=a_{22}^{+}(0)=1$$

*are the Wiener–Hopf factorization of the diagonal elements.*

*If* *ν*_2_ ≤ *ν*_1_ + 1, *then*

$$\rho_{1}=\nu_{1},\;\rho_{2}=\nu_{2}.$$

*If* *ν*_2_ ≥ *ν*_1_ + 2, *then*

$$\rho_{1}=\nu_{1}+\rho ,\;\rho_{2}=\nu_{2}-\rho .$$

*Here ρ is the rank of the matrix* $T_{\nu }$, *ν* = [(*ν*_1_ + *ν*_2_)/2], *and* $T_{\nu }$ *is the Toeplitz matrix* (3.13) *or* (3.14), *consisting of the Fourier coefficients* $a_{\nu_{1}+1},\ldots ,a_{\nu_{2}-1}$ *of function* (2.2), *i.e.* $a(t)=a_{21}(t)/(a_{11}^{+}(t)a_{22}^{-}(t))$.

**Remark 3.6.**

In order not to complicate the presentation of the results in the theorem, we have used different orderings for the partial indices: if *ν*_2_ ≤ *ν*_1_ − 1 then we assume that *ρ*_1_ ≥ *ρ*_2_, and *ρ*_1_ ≤ *ρ*_2_ in other cases.

**Remark 3.7.**

It follows from theorem 3.5 that the partial indices of a triangular 2 × 2 matrix function can take any intermediate values between the least and greatest values of the indices of diagonal elements. It is interesting that this property is not preserved for higher order triangular matrix functions. It turned out that some of the intermediate values are prohibited [22,23,25].

**Remark 3.8.**

We now know the partial indices of *A*(*t*) and we can list the cases of stability/instability in the sense of GKB. Recall that the indices are GKB-stable iff max*ρ*_*j*_ − min*ρ*_*j*_ ≤ 1. In table 1 we present the dependence of the right partial indices on the indices of the diagonal elements and indicate the cases of stability/instability in the partial indices.

**Table 1.** The dependence of the right partial indices *ρ*_1_, *ρ*_2_ on the indices *ν*_1_, *ν*_2_ of the diagonal elements of triangle matrix *A*(*t*). Here *ρ* = *ν* − *ν*_1_ is the rank of the matrix $T_{\nu }$, where *ν* = [(*ν*_1_ + *ν*_2_)/2], and $T_{\nu }$ is the Toeplitz matrix (3.13) or (3.14).

	*ν*_1_, *ν*_2_	the sequence {*a*_*j*_}	*ρ*_1_	*ρ*_2_	GKB criterion
1	*ν*_2_ ≤ *ν*_1_ + 1		*ν*_1_	*ν*_2_	stable/unstable
2	*ν*_2_ ≥ *ν*_1_ + 2	$a_{\nu_{1}+1}=\cdots =a_{\nu_{2}-1}=0$	*ν*_1_	*ν*_2_	unstable
3	*ν*_2_ = *ν*_1_ + 2	$a_{\nu_{1}+1}\neq 0$	*ν*_1_ + 1	*ν*_1_ + 1	stable
4	*ν*_2_ ≥ *ν*_1_ + 3	the sequence $a_{\nu_{1}+1}^{\nu_{2}-1}$ is non-zero	*ν*_1_ + *ρ*	*ν*_2_ − *ρ*	stable/unstable

In those cases when both options are open with respect to the validity of the GKB criterion, we present below the accurate descriptions.

**Corollary 3.9.**

*The condition* max*ρ*_*j*_ − min*ρ*_*j*_ ≤ 1 *is fulfilled in the following cases only*:

(1a) *ν*_2_ = *ν*_1_ − 1, *then* (*ρ*_1_, *ρ*_2_) = (*ν*_1_ − 1, *ν*_1_),
(1b) *ν*_2_ = *ν*_1_, *then* (*ρ*_1_, *ρ*_2_) = (*ν*_1_, *ν*_1_),
(1c) *ν*_2_ = *ν*_1_ + 1, *then* (*ρ*_1_, *ρ*_2_) = (*ν*_1_, *ν*_1_ + 1),
(3) *ν*_2_ = *ν*_1_ + 2 *and* $a_{\nu_{1}+1}\neq 0,$ *then* (*ρ*_1_, *ρ*_2_) = (*ν*_1_ + 1, *ν*_1_ + 1),
(4a) *ν*_2_ ≥ *ν*_1_ + 3, *ν*_1_ + *ν*_2_ *is even and* $\text{rank}\;T_{\nu }=\nu -\nu_{1},$ *then* (*ρ*_1_, *ρ*_2_) = (*ν*, *ν*), *ν* = (*ν*_1_ + *ν*_2_)/2,
(4b) *ν*_2_ ≥ *ν*_1_ + 3, *ν*_1_ + *ν*_2_ *is odd and* $\text{rank}\;T_{\nu }=\nu -\nu_{1},$ *then* (*ρ*_1_, *ρ*_2_) = (*ν*, *ν* + 1), *ν* = (*ν*_1_ + *ν*_2_ − 1)/2.

Thus it is only for these cases that the indices of a triangular 2 × 2 matrix function will be preserved under an *arbitrary* sufficiently small perturbation.

**Example 3.10.**

Let *ν*_2_ = *ν*_1_ + 4. Then the partial indices are completely defined by the three parameters $a_{\nu_{1}+1},$ $a_{\nu_{1}+2},$ $a_{\nu_{1}+3}$ and the matrix *T*_*ν*_ has the form

$$T_{\nu_{1}+2}=\left(\begin{array}{cc} a_{\nu_{1}+2} & a_{\nu_{1}+1}\\ a_{\nu_{1}+3} & a_{\nu_{1}+2} \end{array}\right).$$

By theorem 3.5 we have

$$(\rho_{1},\rho_{2})=\left\{ \begin{array}{ll} \left(\nu_{1}+2,\nu_{1}+2\right) & \text{if }a_{\nu_{1}+2}^{2}-a_{\nu_{1}+1}a_{\nu_{1}+3}\neq 0,\\ \left(\nu_{1}+1,\nu_{1}+3\right) & \text{if }a_{\nu_{1}+2}^{2}-a_{\nu_{1}+1}a_{\nu_{1}+3}=0,\{a_{\nu_{1}+1},a_{\nu_{1}+2},a_{\nu_{1}+3}\}\;\text{is a non-zero sequence}\\ \left(\nu_{1},\nu_{1}+4\right) & \text{if }a_{\nu_{1}+1}=a_{\nu_{1}+2}=a_{\nu_{1}+3}=0. \end{array}\right.$$

The geometric meaning of the above statement in the real case can be given in terms of triples of parameters, $\left(a_{\nu_{1}+1},a_{\nu_{1}+2},a_{\nu_{1}+3}\right)$ being points in the affine space $R^{3}$.

**Figure 1.**
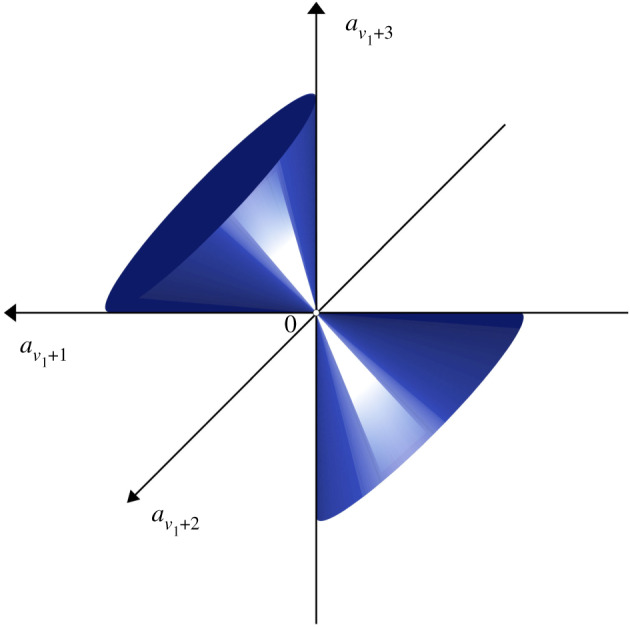
The loci of the partial indices in the affine space $R^{3}$. (Online version in colour.)

— The locus of the points for which (*ρ*_1_, *ρ*_2_) = (*ν*_1_ + 1, *ν*_1_ + 3) is the cone $a_{\nu_{1}+2}^{2}-a_{\nu_{1}+1}a_{\nu_{1}+3}=0$, excluding the vertex.
— The locus for which (*ρ*_1_, *ρ*_2_) = (*ν*_1_, *ν*_1_ + 4) is the vertex of the cone.
— The other points from the space $R^{3}$ correspond to the stable system of the indices: (*ρ*_1_, *ρ*_2_) = (*ν*_1_ + 2, *ν*_1_ + 2) (figure 1).

**Remark 3.11.**

At first glance, this picture contradicts Bojarski’s statement, as discussed in the Introduction. Specifically, it is clear that the set of matrices with common partial indices—(*ρ*_1_, *ρ*_2_) = (*ν*_1_ + 2, *ν*_1_ + 2) (domains inside and outside of the cone)—is not connected. Since the parameters are real, we could represent the geometrical structure in three-dimensional real space. However, Bojarski’s result deals with three-dimensional complex space, in which the respective domain is clearly connected.

## 4. Preserving/changing partial indices upon perturbation in the class $T^{2\times 2}$

In §3 we constructed the right Wiener–Hopf factorization of a triangular 2 × 2 matrix function *A*(*t*) and explicitly obtained its right partial indices (see theorem 3.5). The explicit formulae for the indices allowed us to obtain the effective conditions for the stability of the indices under an *arbitrary* sufficiently small perturbation of *A*(*t*), i.e. the conditions for the GKB stability. These conditions are given in corollary 3.9.

However, in the stability analysis of the factorization of triangular 2 × 2 matrix functions, it is more natural to assume that a perturbation does not remove a matrix function from the given class. This leads to the following definition.

**Definition 4.1.**

Let $A(t)\in GT^{2\times 2}$. The right partial indices of *A*(*t*) are called T-stable if there exists ε > 0 such that any matrix function $\tilde{A}(t)\in T^{2\times 2}$, satisfying the inequality $||A(t)-\tilde{A}(t)||<\varepsilon$, has the same system of right partial indices as *A*(*t*).

We note that for a sufficiently small ε the matrix function $\tilde{A}(t)$ is invertible; hence, it admits the Wiener–Hopf factorization $\tilde{A}(t)={\tilde{A}}_{-}(t)\tilde{D}(t){\tilde{A}}_{+}(t),$ where $\tilde{D}(t)=\text{diag}[t^{{\tilde{\rho }}_{1}},t^{{\tilde{\rho }}_{2}}]$.

The regular perturbations introduced in [18] are obviously T-stable for $A(t)\in GT^{2\times 2}$.

We now address the main question of this section; namely, what happens when we perturb a given triangular matrix with a triangular perturbation. We would like to find out whether new cases of the stability can appear under such perturbations.

**Theorem 4.2.**

*Let* $A(t)\in GTW^{2\times 2}$ *and the indices* *ν*_1_, *ν*_2_ *of its diagonal elements* *a*_11_(*t*), *a*_22_(*t*) *satisfy the inequality* *ν*_2_ ≤ *ν*_1_ + 1. *We denote*

$$\varepsilon =\min\{\min_{t\in T}|a_{11}(t)|,\;\min_{t\in T}|a_{22}(t)|\}.$$

*If a matrix function* $\tilde{A}(t)\in TW^{2\times 2}$ *satisfies the inequality* $||\tilde{A}(t)-A(t)||<\varepsilon$, *then* $\tilde{A}(t)$ *is invertible and has the same partial indices as A*(*t*),

$${\tilde{\rho }}_{1}=\rho_{1}=\nu_{1},\;{\tilde{\rho }}_{2}=\rho_{2}=\nu_{2}.$$

**Proof.**

The proof is straightforward and we present it here only for completeness. By theorem 3.5, for the original matrix function *A*(*t*) we have *ρ*_1_ = *ν*_1_, *ρ*_2_ = *ν*_2_.

It is clear that

$$\max_{t\in T}|{\tilde{a}}_{11}(t)-a_{11}(t)|\leq ||{\tilde{a}}_{11}(t)-a_{11}(t)||\leq ||\tilde{A}(t)-A(t)||<\min_{t\in T}|a_{11}(t)|=\frac{1}{\max_{t\in T}|a_{11}^{-1}(t)|}.$$

Let us introduce

$$w(t)=1+\frac{{\tilde{a}}_{11}(t)-a_{11}(t)}{a_{11}(t)},\;t\in T,$$

and consider

$${\tilde{a}}_{11}(t)=a_{11}(t)w(t),$$

where we observe that $\max_{t\in T}|w(t)-1|<1$ by the previous inequality. Hence ${\tilde{a}}_{11}(t)\neq 0$ on T. Moreover,

$${\tilde{\nu }}_{1}=\text{ind}\;{\tilde{a}}_{11}(t)=\text{ind}\;a_{11}(t)+\text{ind}\;w(t).$$

Note that ind *w* = 0 since the curve *w*(*t*), t∈T, lies in the disc |*w* − 1| < 1 and does not rotate about the origin. Thus ${\tilde{\nu }}_{1}=\nu_{1}$. Similarly, ${\tilde{a}}_{22}(t)\neq 0$ on T and ${\tilde{\nu }}_{2}=\nu_{2}$.

Therefore, $\tilde{A}(t)$ is invertible and, by theorem 3.5, ${\tilde{\rho }}_{1}=\nu_{1}$, ${\tilde{\rho }}_{2}=\nu_{2}$. □

Thus if *ν*_2_ ≤ *ν*_1_ + 1, then the right partial indices are always T-stable. We see that there are new cases of the stability if we narrow down the class of perturbation.

We now turn to the case *ν*_2_ ≥ *ν*_1_ + 2. From table 1 it follows that if the matrix *T*_*ν*_ is of full rank *ρ* = *ν* − *ν*_1_, then the system of partial indices is GKB-stable and *ρ*_1_ = *ρ*_2_ = *ν* for even *ν*_1_ + *ν*_2_ and *ρ*_1_ = *ν*, *ρ*_2_ = *ν* + 1 for odd. Next, we will prove that this condition is sufficient for T-stability. We will not use the GKB criterion to prove this statement.

**Theorem 4.3.**

*Let* $A(t)\in GTW^{2\times 2}$, *the indices* *ν*_1_, *ν*_2_ *of its diagonal elements* *a*_11_(*t*), *a*_22_(*t*) *satisfy the inequality ν*_2_ ≥ *ν*_1_ + 2, *and the matrix T*_*ν*_ *be of full rank* *ρ* = *ν* − *ν*_1_. *Then there is* ε > 0 *such that any matrix function* $\tilde{A}(t)\in TW^{2\times 2}$ *satisfying the inequality* $||\tilde{A}(t)-A(t)||<\varepsilon$ *is invertible and has the same partial indices as A*(*t*),

$${\tilde{\rho }}_{1}=\rho_{1},\;{\tilde{\rho }}_{2}=\rho_{2}.$$

**Proof.**

First, we choose

$$\varepsilon \leq \min\{\min_{t\in T}|a_{11}(t)|,\;\min_{t\in T}|a_{22}(t)|\}.$$

Then, as in theorem 4.2, the matrix function $\tilde{A}(t)$ is invertible and ${\tilde{\nu }}_{1}=\nu_{1}$, ${\tilde{\nu }}_{2}=\nu_{2}$. We can then apply theorem 3.5 to $\tilde{A}(t)$, and hence

$${\tilde{\rho }}_{1}=\nu_{1}+\tilde{\rho },\;\;{\tilde{\rho }}_{2}=\nu_{2}-\tilde{\rho }.$$

Here $\tilde{\rho }$ is the rank of the matrix ${\tilde{T}}_{\nu }$, consisting of the corresponding Fourier coefficients of the function $\tilde{a}(t)={\tilde{a}}_{21}(t)/(\;{\tilde{a}}_{11}^{+}(t){\tilde{a}}_{22}^{-}(t))$.

Since *T*_*ν*_ is of full rank, there exists an invertible submatrix *S*_*ν*_ of *T*_*ν*_. Let ${\tilde{S}}_{\nu }$ be the submatrix of ${\tilde{T}}_{\nu }$ that is located in the same rows as *S*_*ν*_. We can then estimate $||{\tilde{S}}_{\nu }-S_{\nu }||$. For an *m* × *l* matrix *M*, we employ the norm $||M||=\max_{1\leq j\leq l}\sum_{i=1}^{m}|M_{ij}|$. Since ${\tilde{T}}_{\nu }$, *T*_*ν*_ are Toeplitz matrices generated by the sequences ${\tilde{a}}_{\nu_{1}+1}^{\nu_{2}-1}$, $a_{\nu_{1}+1}^{\nu_{2}-1}$, the following inequalities hold:

$$||{\tilde{S}}_{\nu }-S_{\nu }||\leq ||{\tilde{T}}_{\nu }-T_{\nu }||\leq ||{\tilde{a}}_{\nu_{1}+1}^{\nu_{2}-1}-a_{\nu_{1}+1}^{\nu_{2}-1}||\leq ||\tilde{a}(t)-a(t)||.$$

If a scalar function f(t)∈W(T) admits the Wiener–Hopf factorization *f*(*t*) = *f*_−_(*t*)*t*^*ν*^*f*_+_(*t*), *f*_+_(0) = 1, then the factors *f*_±_(*t*) and their inverses continuously depend on *f*(*t*). Hence, for the factors $a_{11}^{+}(t)$, $a_{22}^{-}(t)$ there exist constants *C*_1_, *C*_2_ such that

$$\left||\frac{1}{{\tilde{a}}_{11}^{+}(t)}-\frac{1}{a_{11}^{+}(t)}\right||\leq C_{1}||{\tilde{a}}_{11}(t)-a_{11}(t)||\leq C_{1}||\tilde{A}(t)-A(t)||,$$

$$\left||\frac{1}{{\tilde{a}}_{22}^{-}(t)}-\frac{1}{a_{22}^{-}(t)}\right||\leq C_{2}||{\tilde{a}}_{22}(t)-a_{22}(t)||\leq C_{2}||\tilde{A}(t)-A(t)||.$$

Therefore, there exists a constant *C*_3_ such that $||\tilde{a}(t)-a(t)||\leq C_{3}||\tilde{A}(t)-A(t)||$ and we obtain the final estimate

$$||{\tilde{S}}_{\nu }-S_{\nu }||\leq C_{3}||\tilde{A}(t)-A(t)||.$$

Now let

$$\varepsilon \leq \min\left\{\min_{t\in T}|a_{11}(t)|,\;\min_{t\in T}|a_{22}(t)|,\;\frac{1}{C_{3}||S_{\nu }^{-1}||}\right\}.$$

In this case, if $||\tilde{A}(t)-A(t)||<\varepsilon$, then the matrix ${\tilde{S}}_{\nu }$ is invertible, and ${\tilde{T}}_{\nu }$ is of full rank $\tilde{\rho }=\rho =\nu -\nu_{1}$, and thus ${\tilde{\rho }}_{1}=\rho_{1}$, ${\tilde{\rho }}_{2}=\rho_{2}$. □

Here we have proved that the inequality max*ρ*_*j*_ − min*ρ*_*j*_ ≤ 1 is sufficient for $T^{2\times 2}$-stability of the partial indices. We note that the proof does not use the GKB criterion and that this condition is effective.

Now we will consider the case of a *T*_*ν*_ of incomplete rank.

**Theorem 4.4.**

*Let* $A(t)\in GTW^{2\times 2}$, *the indices* *ν*_1_, *ν*_2_ *of its diagonal elements* *a*_11_(*t*), *a*_22_(*t*) *satisfy the inequality* *ν*_2_ ≥ *ν*_1_ + 2, *and the rank* *ρ* *of the matrix* *T*_*ν*_ *be such that the inequalities* 0 ≤ *ρ* < *ν* − *ν*_1_ *are fulfilled*.

*Then for any sufficiently small* ε > 0 *and for any* *r*, *ρ* ≤ *r* ≤ *ν* − *ν*_1_, *there exists a matrix function* $\tilde{A}(t)\in GTW^{2\times 2}$ *such that* $||\tilde{A}(t)-A(t)||<\varepsilon$ *and the partial indices of* $\tilde{A}(t)$ *are*

$${\tilde{\rho }}_{1}=\nu_{1}+r,\;{\tilde{\rho }}_{2}=\nu_{2}-r.$$

*Matrix functions* $\tilde{A}(t)$ *with other partial indices* ${\tilde{\rho }}_{1}$, ${\tilde{\rho }}_{2}$ *do not exist in the ε-neighbourhood of A*(*t*).

**Proof.**

Let $\varepsilon \leq \min\{\min_{t\in T}|a_{11}(t)|,\;\min_{t\in T}|a_{22}(t)|\}$ and $||\tilde{A}(t)-A(t)||<\varepsilon$. Then, as above, $\tilde{A}(t)$ is invertible, ${\tilde{\nu }}_{1}=\nu_{1}$, ${\tilde{\nu }}_{2}=\nu_{2}$, and ${\tilde{\rho }}_{1}=\nu_{1}+\tilde{\rho },\;\;{\tilde{\rho }}_{2}=\nu_{2}-\tilde{\rho },$ where $\tilde{\rho }=\text{rank}\;{\tilde{T}}_{\nu }$. As in theorem 4.3 we have

$$||{\tilde{T}}_{\nu }-T_{\nu }||\leq C_{3}||\tilde{A}(t)-A(t)||.$$

The rank of any matrix under a sufficiently small perturbation can only increase. Hence $\tilde{\rho }\geq \rho$, ${\tilde{\rho }}_{1}\geq \rho_{1}$ and ${\tilde{\rho }}_{2}\leq \rho_{2}$ if $||\tilde{A}(t)-A(t)||<\varepsilon$ for sufficiently small ε.

First we prove that for the Toeplitz matrix $T_{\nu }$ generated by the sequence $a_{\nu_{1}+1}^{\nu_{2}-1}$, which is of incomplete rank *ρ*, it is possible to choose the sequence $\delta_{\nu_{1}+1}^{\nu_{2}-1}$ such that, for ${\tilde{a}}_{\nu_{1}+1}^{\nu_{2}-1}=a_{\nu_{1}+1}^{\nu_{2}-1}+\delta_{\nu_{1}+1}^{\nu_{2}-1}$, the corresponding matrix ${\tilde{T}}_{\nu }$ has rank *r*, *ρ* < *r* ≤ *ν* − *ν*_1_.

Let *r* = *ρ* + 1. If *ρ* = 0, that is, $a_{\nu_{1}+1}^{\nu_{2}-1}$ is a zero sequence, then it is sufficient to put $\delta_{\nu_{1}+1}^{\nu_{2}-1}=\{\delta_{\nu_{1}+1},0,\ldots ,0\}$, $\delta_{\nu_{1}+1}\neq 0$.

Now let *ρ* > 0 and *S*_*ρ*_ be an invertible *ρ* × *ρ* submatrix of $T_{\nu }$. Suppose that *S*_*ρ*_ consists of the elements of $T_{\nu }$ on the intersection of the rows with number $i_{1},\ldots ,i_{\rho }$ and the columns with numbers $j_{1},\ldots ,j_{\rho }$,

$$S=T_{\nu }\left(\begin{array}{c} i_{1}\;\ldots \;i_{\rho }\\ j_{1}\;\ldots \;j_{\rho } \end{array}\right).$$

Let $i\notin \{i_{1},\ldots ,i_{\rho }\}$, $j\notin \{j_{1},\ldots ,j_{\rho }\}$ and *S*_*ρ*+1_ be the bordering submatrix $T_{\nu }\left(\begin{array}{c} i_{1}\;\ldots \;i_{\rho },i\\ j_{1}\;\ldots \;j_{\rho },j \end{array}\right)$. The entry in the *i*th row and *j*th column of this matrix is *a*_*ν*+*i*−*j*_. In *T*_*ν*_ we replace this element with ${\tilde{a}}_{\nu +i-j}=a_{\nu +i-j}+\delta_{\nu +i-j}$ and denote the new matrix by ${\tilde{T}}_{\nu }^{1}$ and the new submatrix by ${\tilde{S}}_{\rho +1}$. Obviously, $\det{\tilde{S}}_{\rho +1}=\det S_{\rho +1}+\delta_{\nu +i-j}\det S_{\rho }=\delta_{\nu +i-j}\det S_{\rho }\neq 0$ if *δ*_*ν*+*i*−*j*_ ≠ 0. It is not difficult to verify that all bordering submatrices of ${\tilde{S}}_{\rho +1}$ in ${\tilde{T}}_{\nu }^{1}$ are singular. This means that $\text{rank}\;{\tilde{T}}_{\nu }^{1}$ is equal to *ρ* + 1.

Continuing this process and replacing *ρ* − *r* elements of the sequence $a_{\nu_{1}+1}^{\nu_{2}-1}$, we obtain the matrix ${\tilde{T}}_{\nu }$, which is of rank *r*.

Now let us consider the matrix function

$$\tilde{A}(t)=A(t)+\left(\begin{array}{cc} 0 & 0\\ a_{11}^{+}(t)a_{22}^{-}(t)\sum_{j=\nu_{1}+1}^{\nu_{2}-1}\delta_{j}t^{j} & 0 \end{array}\right).$$

If we choose the sequence $\delta_{\nu_{1}+1}^{\nu_{2}-1}$ such that $\sum_{j=\nu_{1}+1}^{\nu_{2}-1}|\delta_{j}|<\frac{\varepsilon }{||a_{11}^{+}||||a_{22}^{-}||}$, then $||\tilde{A}(t)-A(t)||<\varepsilon$, and with respect to the matrix function $\tilde{A}(t)$ the matrix ${\tilde{T}}_{\nu }$ has a rank equal to the given *r*. Thus ${\tilde{\rho }}_{1}=\nu_{1}+r,\;{\tilde{\rho }}_{2}=\nu_{2}-r$. □

It follows from theorems 4.3 and 4.4 that, in the case of *ν*_2_ ≥ *ν*_1_ + 2, the partial indices of *A*(*t*) are stable iff the matrix $T_{\nu }$ is of full rank. In this case *ρ*_1_ = *ρ*_2_ = *ν* or *ρ*_1_ = *ν*, *ρ*_2_ = *ν* + 1.

**Remark 4.5.**

We have proved that, in the case of *ν*_2_ ≤ *ν*_1_ + 2, the partial indices of $A(t)\in GTW^{2\times 2}$ are completely defined by the finite number of the parameters $a_{\nu_{1}+1},\ldots ,a_{\nu_{2}-1}$. Let us suppose that we have *a priori* information about the values of these Fourier coefficients for a certain matrix function *A*(*t*), and that we can guarantee that some perturbations will preserve these values. Then under these perturbations the matrix function $\tilde{A}(t)$ has the same indices as *A*(*t*).

**Example 4.6.**

Let the factorizations of the diagonal elements have the form

$$a_{11}=t^{\nu_{1}}a_{11}^{-},\;a_{22}=a_{22}^{+}t^{\nu_{1}+2}$$

and $a_{21}=t^{\nu_{1}+2}c(t)$, where *c*(*t*) ∈ *W*_+_. Consider the following class of triangular matrix functions:

$$A(t)=\left(\begin{array}{cc} t^{\nu_{1}}a_{11}^{-} & 0\\ t^{\nu_{1}+2}c(t) & a_{22}^{+}t^{\nu_{1}+2} \end{array}\right).$$

Then the indices of *A* are *ρ*_1_ = *ν*_1_, *ρ*_2_ = *ν*_1_ + 2 and they are stable under small perturbations from this class.

Summarizing the results of this section, we can conclude that the conditions for stability of the partial indices in the class $GTW^{2\times 2}$ are weaker than the GKB criterion in the general space $GW^{2\times 2}$, and can be formulated as follows.

**Corollary 4.7.**

*A small perturbation* $\tilde{A}\in GTW^{2\times 2}$ *of the matrix* $A\in GTW^{2\times 2}$ *preserves its right partial indices* (${\tilde{\rho }}_{j}=\rho_{j}$, *j* = 1, 2) *in the following cases* (*the values of ρ*_*j*_ *are collected in* *table 1*):

(1) *ν*_2_ ≤ *ν*_1_ + 1,
(3) *ν*_2_ = *ν*_1_ + 2 *and* $a_{\nu_{1}+1}\neq 0,$
(4a) *ν*_2_ ≥ *ν*_1_ + 3 *and* $\text{rank}\;T_{\nu }=\nu -\nu_{1},$
(4b) *ν*_2_ ≥ *ν*_1_ + 3, $0\leq \text{rank}\;T_{\nu }<\nu -\nu_{1}$ *and* $\text{rank}\;{\tilde{T}}_{\nu }=\text{rank}\;T_{\nu }$.

## 5. Discussion and conclusion

In our paper we have discussed the behaviour of the partial indices of a given matrix function *A*(*t*) under perturbations from the ε-neighbourhood in different classes of matrix functions. We consider both stable and unstable configurations of the partial indices. Our study is restricted to a specific class of triangular matrix functions given on the unit circle with entries from the Wiener algebra. Even in this case, when the factorization technique is well developed, the structure of the parametric space (guiding the types of matrix perturbations) is non-trivial.

The developed approach can be applied for matrices of a larger order as well as for those belonging to different classes of matrix functions. We choose here the case of triangular 2 × 2 matrices with Wiener entries since in this case it is possible to determine an explicit factorization and thus illustrate the main ideas by simple examples.

We have shown, in particular, that, when the orders *ν*_1_, *ν*_2_ of the diagonal elements of an initial matrix *A*(*t*) satisfy the condition *ν*_2_ ≤ *ν*_1_ + 1, for any small perturbation $\tilde{A}(t)\in T^{2\times 2}$ from a certain small neighbourhood of *A*(*t*) the partial indices remain the same.

It follows from theorems 4.3 and 4.4 that for *ν*_2_ ≥ *ν*_1_ + 2 the corresponding partial indices are stable iff the Toeplitz matrix $T_{\nu }$ is of full rank. We note that the proof of this fact is given without appealing to the GKB criterion. In this case, we have also singled out a class of perturbation which yields the preservation of partial indices: if we have preliminary information on the values of the Fourier coefficients $a_{\nu_{1}+1},\ldots ,a_{\nu_{2}-1}$ for the matrix *A*(*t*), then those perturbations of *A*(*t*) that preserve these values lead to the matrix $\tilde{A}(t)$, with identical partial indices to those in *A*(*t*).

Note that, when computing an approximate factorization, the algorithm should be naturally designed in the direction of selecting the approximate factors in a unique way. For example, one might want to prescribe the values of the factors at a certain point. Thus, in formula (3.4), the plus-factor would be the unit matrix at *z* = 0 for *k* = 1, but the minus-factor would be the unit matrix at *z* = ∞ for *k* = 2. It can be directly checked that, under one of these conditions, (*A*_0+_(0) = *I* or *A*_0−_(∞) = *I*) we get a unique factorization in the case *ν*_1_ = *ν*_2_. Surely these conditions can be achieved (e.g. [26]). A detailed discussion on possible uniqueness conditions is to follow.

Note also that an extension of our results to higher order triangular matrix functions is not trivial and requires development of a constructive factorization. Primachuk & Rogosin [6] have suggested an inductive approach to the transition from triangular 2 × 2 matrix functions to a higher order one, based on the Chebotarev method. Another inductive approach was proposed in [21]. The factorization problem for higher order triangular matrix functions has been reduced to the factorization problem for analytic matrix functions. In turn, the latter problem can be explicitly solved by the method of essential polynomials. However, a stability analysis of the problem for the analytic matrix functions is still under development.

## Footnotes

1 The constructive procedure to determine such conditions in the general case is still unknown.

## Data accessibility

This article does not contain any additional data.

## Appendix A. The technique of essential indices and essential polynomials in the factorization problem

**(a) Essential indices and essential polynomials of a sequence**

Let *M*, *N* be integers, *M* < *N*, and $c_{M}^{\;N}=(c_{M},c_{M+1},\ldots ,c_{N})$ be a non-zero sequence of complex numbers. In this section we introduce notions of essential indices and essential polynomials for the sequence $c_{M}^{\;N}$. These notions were given in a more general setting in [20]. Here we will consider the scalar case only, and thus the complicated considerations in [20] can be greatly simplified.

Let us form the family of Toeplitz matrices

$$T_{k}(c_{M}^{\;N})={\left(c_{i-j}\right)}_{\begin{array}{c} \;i=k,k+1,\ldots ,N\\ j=0,1,\ldots ,k-M \end{array}},\;M\leq k\leq N,$$

and study the sequence of the spaces $\ker T_{k}(c_{M}^{\;N})$, *M* ≤ *k* ≤ *N*. Further, it is more convenient to deal not with vectors $Q={\left(q_{0},q_{1},\ldots ,q_{k-M}\right)}^{t}\in \ker\;T_{k}$ but with their generating polynomials *Q*(*z*) = *q*_0_ + *q*_1_ *z* + · · · + *q*_ *k*−*M*_ *z*^*k*−*M*^. We will use the spaces $N_{k}$ of the generating polynomials instead of the spaces $\ker T_{k}$. The generating function $\sum_{j=M}^{N}c_{k}z^{k}$ of the sequence $c_{M}^{\;N}$ will be denoted by $c_{M}^{\;N}(z)$.

Let us introduce a linear functional *σ* by the formula: *σ*{*z*^*j*^} = *c*_−*j*_, −*N* ≤ *j* ≤ −*M*. The functional is defined on the space of rational functions of the form $Q(z)=\sum_{j=-N}^{-M}q_{j}z^{j}.$ In the theory of orthogonal polynomials it is called the Stieltjes functional. Besides this algebraic definition of *σ* we will use the following analytic definition:

A 1 $$\sigma \{Q(z)\}=\frac{1}{2\pi i}\int_{\Gamma }t^{-1}c_{M}^{\;N}(t)Q(t)\;\text{d}t.$$

Here *Γ* is any closed contour around the point *z* = 0.

Denote by $N_{k}\;(M\leq k\leq N)$ the space of polynomials *Q*(*z*) with the formal degree *k* − *M*, satisfying the orthogonality conditions:

A 2 $$\sigma \{z^{-i}Q(z)\}=0,\;i=k,k+1,\ldots ,N.$$

From definition (A 1) it follows that *σ*{*z*^−*i*^*Q*(*z*)} is the coefficient in *z*^*i*^ in the Laurent expansion of $c_{M}^{N}(z)Q(z)$. Hence the conditions (2) mean that in the expansion of $c_{M}^{N}(z)Q(z)$ there exists a lacuna, that is, the coefficients in *z*^*i*^ for *i* = *k*, …, *N* are equal to zero. This fact will be used later.

It is easily seen that $N_{k}$ is the space of generating polynomials of the vectors in $\ker T_{k}$. For convenience, we put $N_{M-1}=0$ and denote by $N_{N+1}$ the (*N* − *M* + 2)-dimensional space of all polynomials with formal degree *N* − *M* + 1. If necessary, the more detailed notation $N_{k}(c_{M}^{\;N})$ is used instead of $N_{k}$.

Let *d*_*k*_ be the dimension of the space $N_{k}$ and Δ_*k*_ = *d*_*k*_ − *d*_*k*−1_ (*M* ≤ *k* ≤ *N* + 1). The following proposition is crucial for further considerations.

Proposition A.1. *For any non-zero sequence* $c_{M}^{N}$ *the following inequalities*

A 3 $$0=\Delta_{M}\leq \Delta_{M+1}\leq \cdots \leq \Delta_{N}\leq \Delta_{N+1}=2$$

*are fulfilled*.

Proof. It follows from definition (2) that $N_{k}$ and $zN_{k}$ are subspaces of $N_{k+1}$. Let us find $\dim(N_{k}+zN_{k})$. To do this, we will prove that

$$N_{k}⋂zN_{k}=zN_{k-1}.$$

Indeed, a polynomial *Q*(*z*) belongs to $N_{k}⋂zN_{k}$ iff *Q*(*z*) = *zQ*_1_(*z*), where $\deg Q_{1}(z)\leq k-M-1$, *σ*{*z*^−*i*^*Q*_1_(*z*)} = 0, for *i* = *k*, *k* + 1, …, *N*, and *σ*{*z*^−*i*^*zQ*_1_(*z*)} = 0, for *i* = *k*, *k* + 1, …, *N*. This precisely means that $Q_{1}(z)\in N_{k-1}$. Then by the Grassman formula we have

$$\dim(N_{k}+zN_{k})=\dim N_{k}+\dim(zN_{k})-\dim N_{k}⋂zN_{k}=2d_{k}-d_{k-1}.$$

Hence

$$\dim N_{k+1}-\dim(N_{k}+zN_{k})=\Delta_{k+1}-\Delta_{k}\geq 0.$$

 □

It follows from inequalities (3) that there exist integers *μ*_1_ ≤ *μ*_2_ such that

4 $$\begin{array}{rl} & \Delta_{M}=\ldots =\Delta_{\mu_{1}}=0,\\ & \Delta_{\mu_{1}+1}=\ldots =\Delta_{\mu_{2}}=1\\ \text{and} & \Delta_{\mu_{2}+1}=\ldots =\Delta_{N+1}=2. \end{array}\}$$

If the second row of these relations is absent, we assume *μ*_1_ = *μ*_2_.

Definition A.2. The integers *μ*_1_, *μ*_2_ will be called *essential indices* of the sequence $c_{M}^{\;N}$.

Proposition A.3. *For any non-zero sequence* $c_{M}^{N}$ *we have*

$$\mu_{1}+\mu_{2}=M+N.$$

Proof. From the definition of the differences Δ_*k*_ we have

$$\sum_{k=M}^{N+1}\Delta_{k}=d_{N+1}=N-M+2.$$

On the other hand, it follows from (4) that

$$\sum_{k=M}^{N+1}\Delta_{k}=\mu_{2}-\mu_{1}+2(N-\mu_{2}+1).$$

Comparison of these relations gives *μ*_1_ + *μ*_2_ = *M* + *N*. □

The following proposition provides formulae for the essential indices.

Proposition A.4. *Let* *ρ* = rank *T*_[(*N*+*M*)/2]_, *where* [(*N* + *M*)/2] *is the integral part of* (*N* + *M*)/2. *Then the essential indices* *μ*_1_, *μ*_2_ *are found by the formulae*

A 5 $$\mu_{1}=M+\rho -1,\;\mu_{2}=N-\rho +1.$$

Proof. Let us denote *ν* = [(*M* + *N*)/2]. Since *μ*_1_ + *μ*_2_ = *M* + *N*, we have *μ*_1_ ≤ *ν* ≤ *μ*_2_. If *μ*_1_ = *ν*, then $d_{\nu }=0$, that is, the matrix $T_{\nu }$ is of full rank: *ρ* = *ν* − *M* + 1. This means that *μ*_1_ = *ρ* + *M* − 1, hence *μ*_2_ = *N* − *ρ* + 1. Now let *μ*_1_ < *ν* ≤ *μ*_2_. From (4) we have

$$d_{\nu }=\sum_{k=M}^{\nu }\Delta_{k}=\nu -\mu_{1},$$

that is, *ν* − *M* + 1 − *ρ* = *ν* − *μ*_1_. Formulae *μ*_1_ = *ρ* + *M* − 1, *μ*_2_ = *N* − *ρ* + 1 once again hold. □

As noted above, $N_{k}$ and $zN_{k}$ are subspaces of $N_{k+1}$, *M* − 1 ≤ *k* ≤ *N*. Let *h*_*k*+1_ be the dimension of any complement $H_{k+1}$ of the subspace $N_{k}+zN_{k}$ in the whole space $N_{k+1}$.

From (4) we see that *h*_*k*+1_ ≠ 0 iff *k* = *μ*_*j*_ (*j* = 1, 2), *h*_*k*+1_ = 1 if *μ*_1_ < *μ*_2_, and *h*_*k*+1_ = 2 for *μ*_1_ = *μ*_2_. Therefore, $N_{k+1}=N_{k}+zN_{k}$ for *k* ≠ *μ*_*j*_, and $N_{k+1}=(N_{k}+zN_{k})⊕H_{k+1}$ for *k* = *μ*_*j*_.

Definition A.5. Let *μ*_1_ = *μ*_2_. Any polynomials *R*_1_(*z*), *R*_2_(*z*) that form a basis for the two-dimensional space $N_{\mu_{1}+1}$ are called the essential polynomials of the sequence $c_{M}^{\;N}$, corresponding to the essential index *μ*_1_ = *μ*_2_. If *μ*_1_ < *μ*_2_, then any polynomial *R*_*j*_(*z*) that is a basis for a one-dimensional complement $H_{\mu_{j}+1}$ is said to be the essential polynomial of the sequence corresponding to the essential index *μ*_*j*_, *j* = 1, 2.

It follows from theorem 4.1 of [20] that in the scalar case the following criterion of essentialness is fulfilled. We will use only the necessary part of the following proposition and we will give the proof only for this part.

Proposition A.6. *Integers* *μ*_1_, *μ*_2_ *such that* *μ*_1_ + *μ*_2_ = *M* + *N* *are the essential indices, and polynomials* $R_{1}(z)\in N_{\mu_{1}+1}$, $R_{2}(z)\in N_{\mu_{2}+1}$ *are the essential polynomials of the sequence* $c_{M}^{N}$ *iff*

$$\sigma_{0}:=\sigma \{z^{-N-1}[R_{2}(0)R_{1}(z)-R_{1}(0)R_{2}(z)]\}\neq 0.$$

*Proof of necessity.* Let *μ*_1_, *μ*_2_ be essential indices and $R_{1}(z)\in N_{\mu_{1}+1}$, $R_{2}(z)\in N_{\mu_{2}+1}$ be the essential polynomials of the sequence. Let us extend this sequence to the right by an arbitrary number *c*_*N*+1_. Then the formula for *σ*_0_ can be rewritten in the form

$$\sigma_{0}=\det\left(\begin{array}{cc} \tilde{\sigma }\{z^{-N-1}R_{1}(z)\} & \tilde{\sigma }\{z^{-N-1}R_{2}(z)\}\\ R_{1}(0) & R_{2}(0) \end{array}\right).$$

Here $\tilde{\sigma }$ is the Stieltjes functional for the extended sequence $c_{M}^{N+1}$.

Suppose that *σ*_0_ = 0. Then there exist constants *α*_1_, *α*_2_, not all zero, such that

A 6 $$\alpha_{1}{\tilde{\sigma }}_{R}\{z^{-N-1}R_{1}(z)\}+\alpha_{2}{\tilde{\sigma }}_{R}\{z^{-N-1}R_{2}(z)\}=0$$

and

A 7 $$\alpha_{1}R_{1}(0)+\alpha_{2}R_{2}(0)=0.$$

Let us introduce the polynomial

$$Q(z)=\alpha_{1}R_{1}(z)+\alpha_{2}R_{2}(z)\in N_{\mu_{2}+1}.$$

From (7) it follows that *Q*(*z*) = *zQ*_1_(*z*) and that the degree of *Q*_1_(*z*) is not greater than *μ*_2_ − *M*, and from (6) it follows that *σ*{*z*^−*N*^*Q*_1_(*z*)} = 0. Since $Q(z)\in N_{\mu_{2}+1}$, we have $Q_{1}(z)\in N_{\mu_{2}}$. Now

A 8 $$\alpha_{2}R_{2}(z)=zQ_{1}(z)-\alpha_{1}R_{1}(z)\in N_{\mu_{2}}+zN_{\mu_{2}}.$$

However, by the definition of the right essential polynomials we have $R_{2}(z)\notin N_{\mu_{2}}+zN_{\mu_{2}}$. Hence condition (8) is fulfilled iff *α*_2_ = 0. By repeating these arguments for the essential index *μ*_1_, we obtain *α*_1_ = 0. This contradiction shows that *σ*_0_ ≠ 0.

**(b) From the essential indices and essential polynomials to the factorization**

We will now obtain the connection between the problem of finding the essential indices and essential polynomials for the sequence $c_{M}^{N}$ and the problem of the Wiener–Hopf factorization of the triangular 2 × 2 matrix function of the special form

A 9 $$P(z)=\left(\begin{array}{cc} z^{M-1} & 0\\ \sum_{i=M}^{N}c_{i}z^{i} & z^{N+1} \end{array}\right),\;z\in \Gamma ,$$

relative to any simple smooth contour *Γ* around 0. Here the sequence $c_{M}^{N}=\{c_{M},\ldots ,c_{N}\}$ is non-zero.

Let *μ*_1_, *μ*_2_ be the essential indices and *R*_1_(*z*), *R*_2_(*z*) be the right essential polynomials of the sequence $c_{M}^{N}$.

For the appropriate essential polynomials $R_{j}(z)\in N_{\mu_{j}+1}$, as indicated above, the Laurent expansion of $c_{M}^{N}(z)R_{j}$ has a lacuna. This yields the following relationship:

A 10 $$c_{M}^{N}(z)R_{j}(z)=z^{\mu_{j}}\alpha_{j}^{-}(z)-z^{N+1}\beta_{j}^{+}(z),\;j=1,2.$$

Here $\alpha_{j}^{-}(z)\;(\beta_{j}^{+}(z))$ is a polynomial in *z*^−1^ (in *z*) with degree that is less than or equal to *μ*_*j*_ − *M*. Now equation (A 10) can be written in the matrix form,

A 11 $$\left(\begin{array}{cc} c_{M}^{N}(z) & z^{N+1} \end{array}\right)\left(\begin{array}{cc} R_{1}(z) & R_{2}(z)\\ \beta_{1}^{+}(z) & \beta_{2}^{+}(z) \end{array}\right)=\left(\begin{array}{cc} \alpha_{1}^{-}(z) & \alpha_{2}^{-}(z) \end{array}\right)\left(\begin{array}{cc} z^{\mu_{1}} & 0\\ 0 & z^{\mu_{2}} \end{array}\right).$$

We denote

$$R_{j}^{-}(z)=z^{-\mu_{j}+M-1}R_{j}(z).$$

Since *R*_*j*_(*z*) has a formal degree in *z* equal to *μ*_*j*_ − *M* + 1, then $R_{j}^{-}(z)$ is a polynomial in *z*^−1^ with the same formal degree. Then from (A 11) we obtain

$$\left(\begin{array}{cc} z^{M-1} & 0\\ c_{M}^{N}(z) & z^{N+1} \end{array}\right)\left(\begin{array}{cc} R_{1}(z) & R_{2}(z)\\ \beta_{1}^{+}(z) & \beta_{2}^{+}(z) \end{array}\right)=\left(\begin{array}{cc} R_{1}^{-}(z) & R_{2}^{-}(z)\\ \alpha_{1}^{-}(z) & \alpha_{2}^{-}(z) \end{array}\right)\left(\begin{array}{cc} z^{\mu_{1}} & 0\\ 0 & z^{\mu_{2}} \end{array}\right).$$

Denote

$${\tilde{P}}_{+}(z)=\left(\begin{array}{cc} R_{1}(z) & R_{2}(z)\\ \beta_{1}^{+}(z) & \beta_{2}^{+}(z) \end{array}\right),\;P_{-}(z)=\left(\begin{array}{cc} R_{1}^{-}(z) & R_{2}^{-}(z)\\ \alpha_{1}^{-}(z) & \alpha_{2}^{-}(z) \end{array}\right),\;D(z)=\left(\begin{array}{cc} z^{\mu_{1}} & 0\\ 0 & z^{\mu_{2}} \end{array}\right).$$

Here ${\tilde{P}}_{+}(z)$ is a matrix polynomial in *z*, $P_{-}(z)$ is a matrix polynomial in *z*^−1^ and

$$P(z){\tilde{P}}_{+}(z)=P_{-}(z)D(z).$$

Let us pass from this equation to its determinants. Since *μ*_1_ + *μ*_2_ = *N* + *M*, we obtain

$$\det{\tilde{P}}_{+}(z)=\det P_{-}(z).$$

By the Liouville theorem we have $\det{\tilde{P}}_{+}(z)=\det P_{-}(z)=\text{const}$. We will now prove that this constant is non-zero. To do this, we find

$${\tilde{P}}_{+}(0)=\left(\begin{array}{cc} R_{1}(0) & R_{2}(0)\\ \beta_{1}^{+}(0) & \beta_{2}^{+}(0) \end{array}\right).$$

From (A 10) we see that $-\beta_{j}^{+}(0)$ is the coefficient of *z*^*N*+1^ in the Laurent expansion of $c_{M}^{N}(z)R_{j}$, that is, $-\tilde{\sigma }\{z^{-N-1}R_{j}(z)\}$. Hence

$${\tilde{P}}_{+}(0)=\left(\begin{array}{cc} R_{1}(0) & R_{2}(0)\\ -\tilde{\sigma }\{z^{-N-1}R_{1}(z)\} & -\tilde{\sigma }\{z^{-N-1}R_{2}(z)\} \end{array}\right)$$

and $\det{\tilde{P}}_{+}(0)=\sigma_{0}\neq 0$ by proposition .6.

Thus, ${\tilde{P}}_{+}(z)$ and $P_{-}(z)$ are unimodular matrix polynomials and

$$P_{+}(z):={\tilde{P}}_{+}^{-1}(z)=\sigma_{0}^{-1}\left(\begin{array}{cc} \beta_{2}^{+}(z) & -R_{2}(z)\\ -\beta_{1}^{+}(z) & R_{1}(z) \end{array}\right).$$

Thus $P(z)=P_{-}(z)D(z)P_{+}(z)$, or, in detail,

A 12 $$P(z)=\sigma_{0}^{-1}\left(\begin{array}{cc} R_{1}^{-}(z) & R_{2}^{-}(z)\\ \alpha_{1}^{-}(z) & \alpha_{2}^{-}(z) \end{array}\right)\left(\begin{array}{cc} z^{\mu_{1}} & 0\\ 0 & z^{\mu_{2}} \end{array}\right)\left(\begin{array}{cc} \beta_{2}^{+}(z) & -R_{2}(z)\\ -\beta_{1}^{+}(z) & R_{1}(z) \end{array}\right),\;z\in \Gamma ,$$

is the Wiener–Hopf factorization of the triangular matrix function P(z) and the essential indices of the sequence $c_{M}^{N}$ are the right partial indices of P(z).
